# Bigels containing different wax-based oleogels as laminating fat replacers in croissants

**DOI:** 10.1016/j.crfs.2025.101042

**Published:** 2025-03-25

**Authors:** Christine Steinkellner, Lina Kroll, Knut Franke

**Affiliations:** Institute of Food and One Health, Leibniz University Hannover, Am Kleinen Felde 30, 30167, Hannover, Germany

**Keywords:** Bigel, Waxes, Solid fat replacer, Laminated pastry, Croissants, Texture

## Abstract

In this study, bigels were developed to mimic the characteristics of traditional laminating fats, butter and margarine, in croissants. The bigels consist of 80 % oleogel (canola oil, wax and monoacylglyceride) and 20 % hydrogel (water and xanthan gum). Beeswax (BW), carnauba wax (CBW), candelilla wax (CLW), and rice bran wax (RBW) were evaluated as oleogelators at concentrations between 12 and 20 % w/w in the oleogel. The effects of wax concentration, temperature, and mechanical work (plasticizing) on texture, solid fat content, and microstructure of the bigels were investigated.

Bigels’ solid fat content and mechanical properties were less temperature sensitive than controls, but mechanical work (plasticizing) had detrimental effects on their texture. Differences in bigel firmness between waxes at the same concentration could be attributed to different wax crystal structures. Plasticized bigels most similar in texture to the controls were those with 18 % BW, 14 % CBW, 14 % CLW, and 20 % RBW. These bigels were tested as laminating fats in croissants at 100 % replacement levels. After lamination, the croissant doughs with bigels exhibited irregular fat layering, resulting in more dense and less airy croissant pore structure. While bigel croissants possessed a comparable volume, they were generally flatter and wider compared to croissants with control fats. In terms of texture, bigel croissants displayed a lower degree of staling, but had overall higher firmness. Furthermore, they had similar springiness and cohesiveness, but increased chewiness. With respect to nutritional value, croissant made with bigels contained significantly less saturated fatty acids.

## Highlights

• Bigels at different wax levels were compared to butter and margarine.
• Mechanical stress decreased bigel firmness by more than 50 %.
• Lower structural integrity of bigels led to inhomogeneous layering in the dough.
• Bigel croissants were denser and firmer, but had similar volume.
• Bigel croissants had improved nutritional value.

## Graphical abstract

**Figure:**
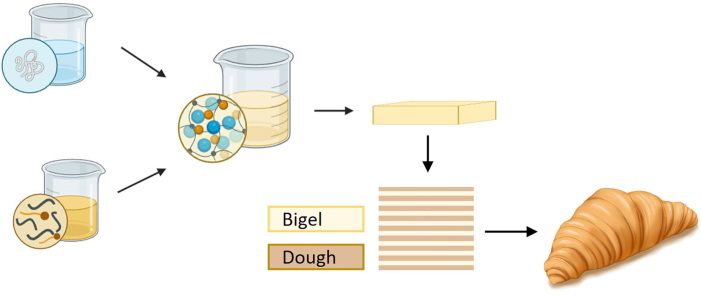

## 1 Introduction

The World Health Organization recommends replacing fats high in saturated fatty acids (SFA) with oils that are rich in mono- or polyunsaturated fatty acids to avoid possible negative health implications of SFA (WHO, 2023). Laminated pastries like croissants are made with up to 35 % laminating fat based on dough weight (Cauvain, 2015; Stauffer and Shahidi, 2005). Traditionally used fats such as butter and margarine are rich in SFAs. In butter, they occur naturally and most margarines contain SFA-rich hydrogenated oils and/or palm fat (Belitz et al., 2009). Hence, replacing those laminating fats with unsaturated oils would improve the nutritional value of the pastries. However, unsaturated oils are generally liquid at room temperature and cannot provide the technological functionality of SFA-rich solid fats (Demirkesen and Mert, 2020; Martins et al., 2018). One approach to modify the physical properties of oils, without compromising the desirable fatty acid composition, is an oil-structuring technique called oleogelation (Silva et al., 2022; Co and Marangoni, 2012). In oleogelation, lipophilic gelling agents immobilize the oil in a self-standing, three dimensional network forming (semi-)solid oleogels (Davidovich-Pinhas, 2018; Doan et al., 2015). Many different types of oleogelators exist, including waxes, fatty alcohols, fatty acids, monoacylglycerides, wax esters, phytosterols or ethylcellulose (Co and Marangoni, 2012; Davidovich-Pinhas et al., 2016). Similarly, water can be structured using hydrophilic gelling agents to form hydrogels (Martins et al., 2023). Polysaccharides, like gums, create a stable soft hydrogel network via hydrogen bonds and ionic interaction (Shakouri et al., 2025). Here, xanthan is often used on account of its low cost and high water binding capacity at low concentrations (Behera et al., 2015; Vershkov and Davidovich-Pinhas, 2023; Fernandes et al., 2023).

Bigels can be formed by emulsifying the two gels under specific shear and temperature conditions (Chao et al., 2024; Shakeel et al., 2021). They are also known as hybrid gels, because they combine the high mechanical strength of oleogels and the viscoelastic properties of hydrogels, outperforming the individual gels (Martins et al., 2023; Chen et al., 2023; Lupi et al., 2017). Hence, bigels can be tailored to fit certain food applications (Chao et al., 2024). Emulsifiers, like monoacylglyceride (MG), can be added to bigels to further improve stability (Nutter et al., 2023a). However, as mentioned above, MG has been shown to act as oleogelator and also as crystallization modifier of wax oleogels (Saffold and Acevedo, 2022; Lupi et al., 2012).

A key factor in adjusting the mechanical properties of bigels to fit certain food applications is the type and concentration of oleogelator (Martins et al., 2023; Fasolin et al., 2021). Natural waxes belong to the most studied group of oleogelators, including candelilla (CLW) (Blake et al., 2014; Toro‐Vazquez et al., 2007; Winkler‐Moser et al., 2019; Hwang et al., 2022), carnauba (CBW) (Blake et al., 2014; Airoldi et al., 2022), rice bran (RBW) (Blake et al., 2014; Hwang et al., 2022) and beeswax (BW) (Winkler‐Moser et al., 2019; Hwang et al., 2022; Abdolmaleki et al., 2022; Yilmaz et al., 2021). The regulatory status for the use of waxes in food varies between the EU and US. All four waxes have FDA approved GRAS status in the US, while only BW, CBW and CLW are approved food additives in the EU (FDA, 2024; European Commission, 2023). The respective regulations may limit the products and levels in which the waxes can be used, but in general, quantum satis applies, which means that only the absolutely necessary quantity should be used. Waxes are not yet specifically approved for oil structuring, but researchers are confident that regulations will be updated once the commercial demand for waxes as oleogelators increases (Scharfe and Flöter, 2020). Depending on the source, waxes contain varying ratios of wax esters, long chain hydrocarbons, free fatty acids, and free fatty alcohol (Blake et al., 2014; Doan et al., 2017). The resulting differences in melting profiles, crystallization behaviour and crystal morphology lead to different gelling behaviour and texture properties of the gels (Doan et al., 2017; Hwang et al., 2012; Wettlaufer and Flöter, 2022; Mattice et al., 2017). Hence, some waxes might be more suitable for certain applications than others. Critical gelling concentrations for the before mentioned waxes have been investigated in numerous studies and generally range between 1 % and 5 % (Blake et al., 2014; Hwang et al., 2012; Doan et al., 2018). This is the minimum amount that is necessary to acquire a soft and stable gel.

However, laminating fats for the production of croissants require a more solid consistency compared to standard baking fats (Stauffer, 1996). Hence, higher wax concentrations are necessary to replace them with oleo- or bigels. During croissant production, the fat is repeatedly sheeted and folded in between layers of yeasted dough until very thin layers have been formed. These layers trap the steam formed in the dough layers during baking, which leads to the desired pore structure and eating properties of croissants (Stauffer and Shahidi, 2005; Ooms et al., 2016). Fat that is too soft would leak out of the dough during sheeting, but fat that is too firm and brittle would puncture through the dough layers (Telloke, 1991a). Therefore, an optimum consistency is required for the formation of the required thin continuous fat layers (Renzetti et al., 2016). In order to make the firm laminating fats more pliable, they are often subjected to plasticizing, or work softening. Thereby, the fat is sheeted and sometimes folded before it is added to the dough (Cauvain, 2015; Telloke, 1991a). The large deformations during sheeting induce structural changes in the fat crystal network altering the consistency of the fat (Renzetti et al., 2016; Kloek et al., 2005). Similarly, the mechanical stress during sheeting has an effect on the structure of bigels, also influencing their consistency (Silva et al., 2022). Compared to pure oleogels, bigels have been shown to have improved recovery after such deformations due to the active filler behaviour of the hydrogel (Vershkov and Davidovich-Pinhas, 2023).

Oleogel technology has been used previously to (partially) replace laminating fat in croissants (Blake and Marangoni, 2015; Espert et al., 2023; Wang et al., 2024). Wax-based oleo- and bigels have been investigated as (partial) fat replacers in bakery products such as gluten free cake (Demirkesen and Mert, 2019), sponge cake (Wettlaufer and Flöter, 2022), cookies (Mert and Demirkesen, 2016a, 2016b; Jang et al., 2015; Pang et al., 2022; Nutter et al., 2023b; Quilaqueo et al., 2022; Barragán-Martínez et al., 2022) and puff pastry (Steinkellner et al., 2024). However, the effect of different wax concentrations on bigel properties and, especially, the impact of mechanical work during plasticizing have not been investigated yet. The present study deals with the development of bigels, which closely resemble the commercial laminating fats, butter or margarine, with respect to water percentage and firmness. Hydrogel-in-oleogel (w/o) type bigels consisting of 80 % oleogel (canola oil, wax and monoacylglyceride) and 20 % hydrogel (water and xanthan gum) were investigated. This ratio was chosen to mimic the oil/water ratio of conventional laminating fats. Also, an oleogel: hydrogel ratio of 80 : 20 has been previously shown to result in bigels with similar properties as semi-solid fats used in bakery applications (Nutter et al., 2023a). Four wax types (BW, CLW, CBW and RBW) were evaluated as oleogelators at concentrations between 12 and 20 % in the oleogel. The aim was to find the concentration for each wax at which the bigel consistency most closely resembles that of the commercial laminating fats butter and margarine. Texture, solid fat content and microstructure of bigels were evaluated. Mechanical properties were investigated before and after plasticizing at 10, 15 and 20 °C and compared to commercial laminating butter and margarine. Bigels with similar texture properties as the two control fats at their respective usage temperatures were incorporated in croissant dough at 100 % replacement level. Fat and dough layer distribution was investigated as well as croissant quality parameters, texture and pore structure. Results will provide useful information about bigel behaviour during the lamination process and will help to further develop bigels based on different wax oleogels as solid fat replacers in laminated and other pastry products.

## 2 Materials and methods

### 2.1 Materials

For bigel production, refined canola oil (CO) (Brökelmann + Co – Ölmühle GmbH + Co, Germany) was purchased in bulk at a local supermarket. Refined candelilla wax (CLW) with a melting range of 68–73 °C, white beeswax (BW) with a melting range of 61–66 °C and xanthan gum (XG) were obtained from Manske GmbH (Germany). Rice bran wax (RBW) with a melting range of 79–85 °C was purchased from DistrEbution GmbH (Germany). Distilled monoacylglyceride (MG) made from edible, fully hydrogenated rapeseed oil (DIMODAN® HR KOSHER No. 1256660, DANISCO, Denmark) and carnauba wax (CBW) with a melting range of 82–86 °C were kindly provided by SENNA Nahrungsmittel GmbH & Co KG (Austria).

For croissant production, wheat flour type 550 with added ascorbic acid was purchased at a local bakery. Sugar and table salt were purchased at a local supermarket. Instant yeast (Instaferm® 01) was obtained from Lallemand Baking (Portugal). Control laminating fats, butter (Art. Nr. 1413254) and margarine (Art. Nr. 1213205), were kindly provided by SENNA Nahrungsmittel GmbH & Co KG (Austria). All raw materials were stored at room temperature (20 ± 2 °C) except control laminating fats, which were stored under refrigerated conditions (6 ± 2 °C) until use.

Lugol solution containing approx. 3.3 g/l iodine und 6.7 g/l potassium iodide to stain the dough layers was purchased from Carl Roth (Karlsruhe, Germany).

### 2.2 Experimental design

In the first set of experiments, oleogels were prepared with CLW, CBW, RBW and BW at concentrations of 12, 14, 16, 18, 20 % w/w related to the oleogel weight. Bigels were prepared with 80 % oleogel and 20 % hydrogel to mimic the oil/water ratio of commercial laminating fats. XG was added as hydrogelator at 1 % w/w related to the hydrogel mass. MG was added as emulsifier at 1 % w/w related to the total bigel mass. Concentrations were selected according to preliminary studies (data not published). Samples were labelled according to wax type and concentration (e.g. CLW12 for candelilla wax at 12 % w/w in the oleogel).

Bigels, which showed similar textural properties after plasticizing as the commercial control fats, butter and margarine, were selected for application in croissants (see 3.1.1). A total of 300 g bigel was produced for each experiment. Experiments were performed in duplicate.

### 2.3 Bigel preparation

Bigel preparation was based on the method described by Vershkov and Davidovich-Pinhas (2023) with modifications.

Oleogels were prepared by combining CO, wax, and MG. The mix was heated under constant magnetic stirring (MR Hei-Tec, Heidolph, Germany) at 400 rpm to the respective wax melting temperature (Table 1). This temperature was held for 10 min to ensure that all components of the wax were completely melted. Then, the oleogels were conditioned to the respective homogenization temperature (Table 1) in a drying chamber (Heratherm OGS100, Thermo Scientific, Germany). Optimal homogenization temperatures for each wax were determined in preliminary tests (data not published). Oleogels had to be liquid enough to ensure emulsification with the hydrogel without too much air bubble formation, but cool enough to ensure fast solidification of the bigel after homogenization without phase separation. Hydrogels were prepared by dispersing XG in distilled water under agitation at 900 rpm and heating up to the respective homogenization temperature (Table 1) using the same heater. Bigels were prepared by combining hydrogel and oleogel followed by homogenization at 20,000 rpm for 3 min using an Ultra Turrax laboratory homogenizer T25 Basic (IKA, Germany) equipped with a pre-heated shearing device (diameter of 25 mm). Afterwards, the resulting bigels were immediately poured into a 170 × 120 × 20 mm mold lined with baking paper. Bigels were allowed to cool and set quiescently at room temperature (20 ± 2 °C) for 1 h before demoulding and were then stored refrigerated (6 ± 2 °C) over night. The cooling rate was approx. 1 °C/min in the center of the bigel mold. The low cooling rate was selected in order to obtain a more elastic gel, as discussed by Co and Marangoni (2012). Resulting bigel plates had a height of 15 ± 2 mm resembling the shape and thickness of commercial laminating fat blocks.

**Table 1:** Melting and homogenization temperatures for oleogels and bigels with different waxes.

Wax	Oleogel melting temperature (°C)	Bigel homogenization temperature (°C)
**Beeswax**	67 ± 2	55 ± 2
**Carnauba wax**	86 ± 2	65 ± 2
**Candelilla wax**	75 ± 2	55 ± 2
**Rice bran wax**	85 ± 2	75 ± 2

### 2.4 Croissant preparation

Croissants were prepared using a basic yeasted dough (see below) together with 50 % w/w laminating fat based on flour weight or approx. 30 % w/w based on dough weight.

For conditioning the laminating fats prior to use, 250 g of bigel or control fat were taken out of refrigeration and left at room temperature (20 ± 2 °C) for 1 h to equilibrate. Fats were then plasticized between two silicone baking mats to a final thickness of 8 mm in 1 mm steps using a reversible sheeter (Kombi SK067, Seewer Rondo, Switzerland). Fat sheets obtained were then refrigerated again for 30 min.

Meanwhile, the base dough was prepared by mixing 1000 g flour with 500 g water, 120 g sugar, 20 g salt and 20 g instant yeast for 2 min on low speed (750 rpm) followed by 4 min intensive kneading on high speed (1500 rpm) in a spiral mixer (SP24F, DIOSNA, Germany). Dough temperature was controlled to not exceed 24 °C by adjusting water temperature. After kneading, the dough was cut in equal halves of 830 ± 2 g. Each half was re-rounded 20 times before wrapping it in plastic foil and resting in the refrigerator for 20 min.

Before combining laminating fat and dough, temperatures of both components were checked to be in the same range. Otherwise, resting times were adjusted accordingly. Optimum lamination temperatures were 13–15 °C and 18–20 °C for butter and margarine, respectively. Temperatures for processing of bigels were chosen in accordance with the required firmness obtained from bigel characterization (see 3.1.1).

**Fig. 1:**
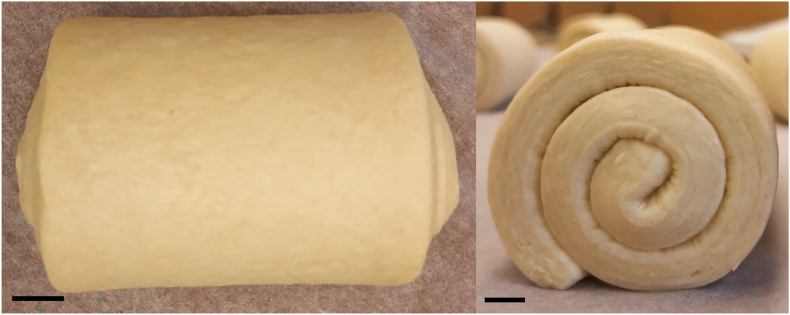
Top (left) and side view (right) of croissants before baking (bar length: 10 mm).

The respective fat was laminated into the dough using the French method (Telloke, 1991b). Firstly, the base dough was sheeted to the same width and double length of the fat sheet. Afterwards, the fat was placed in the center of the dough with the top and bottom being flush and the side pieces of the dough were folded over and sealed manually in the middle. Subsequently the piece was turned 90° and sheeted again to 10 mm thickness by reducing the roller gap by 1 mm after each sheeting. It was then folded in thirds (using the letter fold method), turned 90° and sheeted again. This was repeated three times with 20 min cooling/resting in between. In the end, the theoretical layer count was 55 with 27 fat and 28 dough layers. After the last folding and cooling step, the pastry dough was sheeted to 8 mm thickness and the irregular edges were cut off. The remaining dough was cut into 50 mm × 100 mm rectangles with the longer side being cut in the last sheeting direction to avoid elastic recoil (Bousquieres et al., 2014; Cauvain and Young, 2001). Two rectangles were taken for layer analysis of the dough (see 2.6.1). The remaining rectangles were rolled up into cylinders to resemble “pan au chocolat”, a French pastry made with croissant dough (Fig. 1).

Croissants were put onto a lined baking sheet and then placed in a fermentation chamber (Wiesheu, Germany) at 80 % relative humidity and 32 °C for 60 min (margarine and bigels) or at 28 °C for 90 min (butter). Afterwards, croissants were baked in an industrial deck oven (Wachtel, Germany) for 20 min at 200 °C and 220 °C of bottom and top heat, respectively, including a steam injection at the beginning of baking. Fermentation and baking temperatures were selected according to recommendations by suppliers and experienced bakers. Times were determined in pretrials to gain double the volume during fermentation and to obtain a completely baked, but not burned, pastry. After baking, the pastries were left to cool on a wooden cooling rack for either at least 1 h or overnight depending on the analysis (see below).

### 2.5 Characterization of bigels and control fats

#### 2.5.1 Mechanical properties

**Fig. 2:**
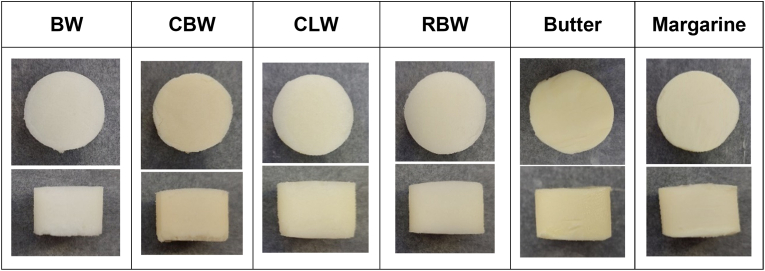
Pictures of fat samples for texture analysis before plasticizing (top and side view). Dimensions: 21.5 mm diameter, 15 mm thickness; BW: beeswax, CBW: carnauba wax, CLW: candelilla wax, RBW: rice bran wax, all presented bigels contained 12 % wax in the oleogel.

After refrigerated storage overnight, bigels as well as control fats were cut into disks with a diameter of 21.5 mm using a circular cutter (Fig. 2). These samples were marked ‘before plasticizing’. To investigate the effect of plasticizing on mechanical properties, the residual plates (bigel or control) were stored at room temperature for 60 min to equilibrate to 20 ± 2 °C. Then, plates were put between silicone baking mats and plasticized. For this purpose, they were sheeted with the sheeter mentioned above to a thickness of 10 mm in steps of 0.5 mm. The resulting plates were folded in half and sheeted again to a thickness of 15 mm using the same step size between sheetings. A similar procedure was applied by Telloke (1991a) for different laminating fats. Afterwards, discs were cut out from the plate and marked ‘after plasticizing’.

Mechanical properties of samples were determined using a Texture Analyser TA HD plus (Stable Micro Systems, United Kingdom) equipped with a P/36R cylindrical probe and a 50 kg load cell. Pre-test speed was set to 1 mm/s, test speed was 2 mm/s, and trigger force was 0.049 N. The compression distance was set to 5 mm, resembling approx. 33 % of 15 mm thickness. The maximum force (N) during compression indicated the firmness and the area under the force-distance curve (N.mm) indicated the total work of plastic deformation also called plasticity (Pădureţ, 2022; Macias-Rodriguez et al., 2020).

The samples were analysed at three temperatures, in the refrigerated state (10 °C) and at the respective application temperatures of butter and margarine, 15 °C and 20 °C. As mentioned above, mechanical bigel properties were determined before and after the plasticizing process. Plasticized samples were analysed 1 h after plasticizing. Samples were analysed at least in triplicate per experimental replication for each type of fat, temperature, and mechanical treatment (n ≥ 6).

#### 2.5.2 Solid fat content

Solid fat content (SFC) of bigels and control fats was measured externally at SENNA Nahrungsmittel GmbH & Co KG (Vienna, Austria) by pulsed nuclear magnetic resonance (pNMR). Sample's SFC was evaluated after tempering to 10, 20 and 30 °C for 30 min using a Bruker Minispec mq-one SFC analyser (Bruker Corporation, USA). Standard deviation for this method was stated as 0.3 %.

#### 2.5.3 Microstructure

A drop of liquid bigel was placed on a preheated microscopy glass slide, topped with a coverslip, and left to cool and solidify at room temperature. Bigel microstructures were observed using an optical microscope with a polarized light filter (SWIFT, SW350T, United States). Pictures were taken at 400x magnification using a digital camera (Canon EOS, 2000D, Canon, Germany).

### 2.6 Characterization of croissants before and after baking

#### 2.6.1 Visualisation of layer distribution in pastry dough

Sample preparation was similar to the method described by Renzetti et al. (2016). Unfermented pastry dough rectangles were cut in half lengthwise into strips of 25 mm × 50 mm and frozen over-night (−18 °C). Then, edges were cut off and, using a razor blade, slices with a thickness of 1 mm were cut and transferred onto a protein-glycerol-coated microscope slide. Slides with the dough layer were dipped into Lugol solution for 60 s to stain the starchy dough, rinsed with 70 % ethanol solution to remove surplus Lugol solution, and let dry for 30 min before the layered structure was examined under a digital microscope (TOMLOV, China).

#### 2.6.2 Evaluation of croissant quality parameters

Volume (V in cm^3^) of baked croissants was evaluated using the rapeseed displacement method after 1 h of cooling. Mass (m in g) as well as dimensions, height (h in mm) and width (w in mm), of the croissants were measured before proofing (h_0_, w_0_) and after baking (m_1_, h_1_, w_1_) using a scale and calliper, respectively. Five croissants were analysed per experimental replication (n = 10). Specific volume (SV), height expansion (HE), and width expansion (WE) were calculated from these data to evaluate croissant properties.

(1) $$\text{SV}=\frac{V}{m_{1}}$$

(2) $$\text{HE}=\frac{h_{1}\;‐\;h_{0}\;}{h_{0}}\;*\;100\;\%$$

(3) $$\text{WE}=\frac{w_{1}\;‐\;w_{0}\;}{w_{0}}\;*\;100\;\%$$

#### 2.6.3 Texture profile analysis of croissants

Texture profile analysis (double compression test) was performed on whole croissants using a Texture Analyser TA HD plus equipped with a P25 cylindrical probe. Speed during the test was 2 mm/s, maximum compression was 50 %, trigger force was 0.049 N, and time between compressions was 5 s.

Croissant firmness (N) was evaluated as the maximum force during first compression, springiness (%) as the ratio between the distance for second and first compression, cohesiveness (%) as the ratio between the areas under the curves of second and first compression and chewiness (−) as firmness x springiness x cohesiveness (Peleg, 2019).

Measurements were conducted after 1 h of cooling (day 0) and one day after baking (day 1) to evaluate possible differences in staling behaviour of croissants for the different formulations. Croissants were stored unsealed at room temperature before measurement to mimic conventional environmental conditions in a bakery. Three croissants were analysed per day and experimental replication (n = 6).

#### 2.6.4 Pore structure evaluation

To evaluate the inner pore structure of the croissants, samples were cut in thirds with a sharp knife and the cross section was stained using acrylic black dye. Then, the stained area of the croissant piece was pressed onto a sheet of white paper to get a picture of the cross section with pores and pore walls. After drying, pictures were scanned and the specific pore surface area was determined based on a stereological method described in Bindrich and Stoyan (1991) and Chiu et al. (2009). In brief, parallel lines were drawn through the image of the structure and the number of transitions from pore wall to pore interior and vice versa were counted for each line. From this number and the real length of the line within the croissant cross section, the specific pore surface can be determined (Chiu et al., 2009). A higher value of this parameter reflects more transitions per length indicating more and smaller pores in the inner structure, whereas a lower number indicates bigger pores and less pore walls. Pore structure evaluation was performed the day after baking. Two croissants were used per experimental replication, three cross sections were analysed per croissant (n = 12).

### 2.7 Statistical analysis

All numeric data were expressed as mean ± standard deviation. Statistical analyses were carried out in RStudio® (Posit Software, PBC) using R version 4.3.3. ANOVA as well as Tukey's test were used to determine statistically significant differences between means. Differences were considered significant at p < 0.05.

## 3 Results and discussion

### 3.1 Fats

#### 3.1.1 Mechanical properties

Commercial laminating fats butter and margarine were used as a benchmark in this study. The firmness of the two control fats at 10, 15 and 20 °C, before and after plasticizing, is shown in Table 2. Firmness of both fats exhibited a strong temperature dependency with significant softening at higher temperatures. The reduced firmness can be attributed to the decreased content of solid triacylglycerides, as the increased temperature melts low-melting fractions (Stauffer, 1996; Truong et al., 2020). This is confirmed by the results of the solid fat content analyses (see chapter 3.1.2).

**Table 2:** Firmness (in N) of butter (B) and margarine (M) at different temperatures before and after plasticizing.

	Before plasticizing			After plasticizing		
	10 °C	15 °C	20 °C	10 °C	15 °C	20 °C
**B**	87.5 ± 12.3a,A∗	21.8 ± 2.7b,A	10.9 ± 1.2c,A	64.5 ± 12.9a,B	13.5 ± 3.1c,B	5.4 ± 0.4d,B
**M**	85.2 ± 6.5a,A	26.3 ± 2.6b,A	20.3 ± 0.3b,A	55.9 ± 10.3a,B	22.8 ± 0.6b,B	14.2 ± 0.7c,B

∗Different lower-case letters indicate significant differences (p < 0.05) between both fats at all temperatures within the same processing state (before or after plasticizing), different upper-case letters indicate significant differences within the same fat and temperature, but different processing states.

According to the supplier, the butter and margarine used in this study exhibit optimal workability at 15 °C and 20 °C, respectively. No significant difference in firmness was measured between butter at 15 °C and margarine at 20 °C, which confirms the recommended processing conditions. The application of mechanical stress during plasticizing significantly lowered the firmness of both butter and margarine. Similar results have been described by Renzetti et al. (2016), who also observed reduced consistency after sheeting of different puff pastry shortening blends. The applied stress induces breakage and partial disintegration of the fat crystal network due the displacement of volume elements during this process, weakening the structure (Kloek et al., 2005).

**Fig. 3:**
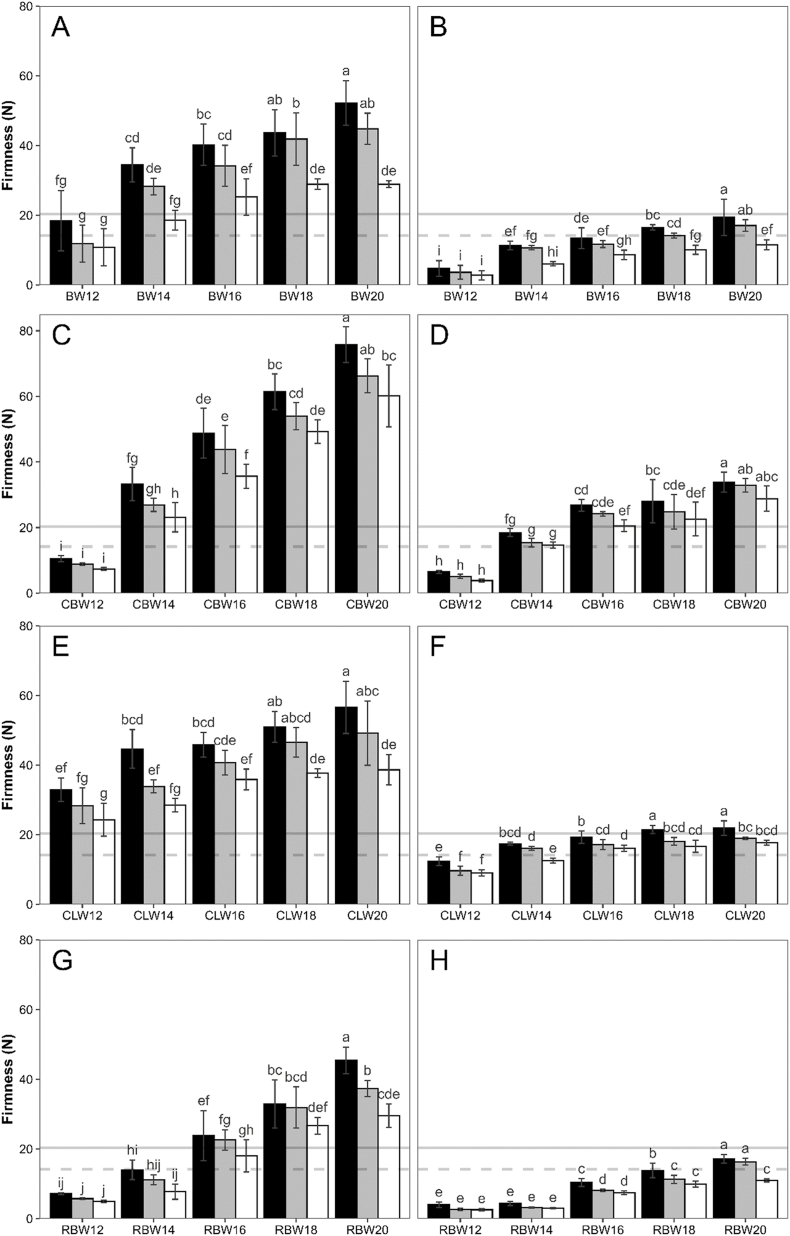
Influence of wax concentration, temperature and plasticizing on firmness of different wax bigels. (A,B) beeswax, (C,D) carnauba wax, (E,F) candelilla wax, (G,H) rice bran wax at wax concentrations between 12 and 20 % in the oleogel; left column before plasticizing, right column after plasticizing; black = 10 °C, grey = 15 °C, white = 20 °C. Reference lines indicate mean firmness of margarine at 20 °C, solid line = unplasticized, dashed line = plasticized. Different lower-case letters indicate significant differences within graphs (p < 0.05).

Fig. 3 depicts firmness results of bigels at concentrations between 12 and 20 % of BW (A,B), CLW (C,D), CBW (E,F) and RBW (G,H) in the oleogel phase. Firmness values of margarine at 20 °C were incorporated in the graphs as reference lines to visualize the target firmness. Because firmness of margarine at 20 °C was very similar to that of butter at 15 °C, the butter firmness was not included in the graphs to ensure clear visibility of the results. All bigels exhibited increased firmness at higher wax concentrations. This is in accordance with previously published studies by Hwang et al. (2022), Jang et al. (2015), Schubert et al. (2022) and Silva et al. (2021) who reported higher oleogel firmness with increasing wax concentration.

**Fig. 4:**
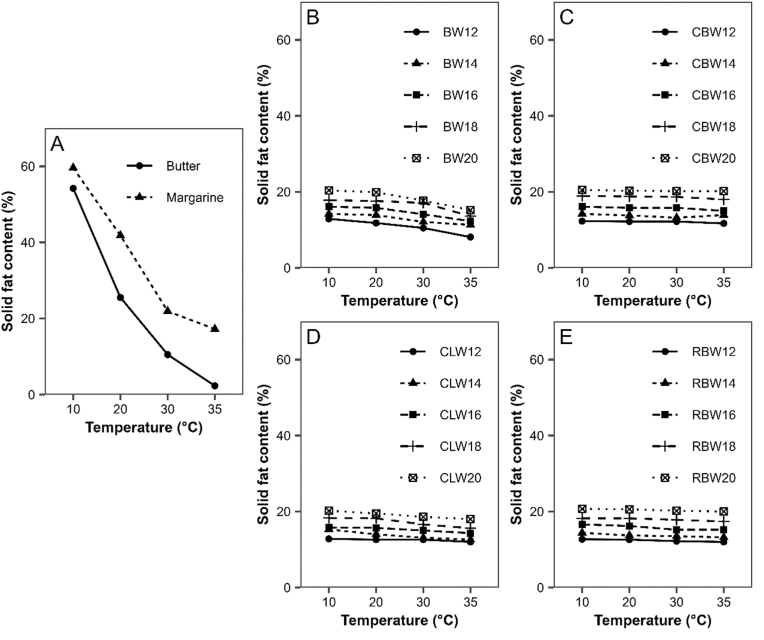
Solid fat content of control fats and bigels at different wax concentrations between 12 and 20 % in the oleogel. (A) butter and margarine, (B) beeswax, (C) carnauba wax, (D) candelilla wax, (E) rice bran wax.

For all waxes and wax concentrations, a reduction of firmness was determined with increasing temperature. A similar trend was discussed by Vershkov and Davidovich-Pinhas (2023) who described reduced firmness of bigels containing candelilla wax oleogel and xanthan hydrogel between 4 and 25 °C. However, in the present study, the reduction was not always significant and firmness did not decrease to the same extend as for butter and margarine (Table 2). This can be attributed to the different type of structuring in the bigels compared to the control fats. The waxy gel structure, which determines the firmness of the oleogels and bigels, had a much higher melting point compared to the solid fat parts in margarine and, especially, butter. Measurement temperatures between 10 and 20 °C are much lower than the melting range of the waxes. Therefore, changes within this temperature range have a lower effect on fat crystal structure and, therefore, mechanical properties. This is confirmed by the data from the solid fat content (Fig. 4), which will be presented and discussed later. Bakers and manufacturers could potentially benefit from the decreased temperature sensitivity of bigels. Cooling steps of the dough during the lamination process could be omitted reducing processing time and energy costs.

Looking at the firmness before and after mechanical work (Fig. 3, left and right column), a huge decrease in firmness of the bigels was detected due to the plasticizing step. Comparing the samples before plasticizing (left columns) with the margarine (lines), most bigels at wax concentrations above 12 % show much higher firmness than the control. Based on these results, bigels with 12 or 14 % wax in the oleogel would be selected as a suitable fit for application in the croissants. However, after plasticizing (right column) bigel samples exhibit a massive decrease in firmness. Bigels that seemed appropriate before plasticizing would be way too soft to be used in the pastries. To reach similar firmness as margarine after plasticizing, higher wax concentrations are required. BW18 at 15 °C, CBW14 at 20 °C, and CLW14 and RBW20 between 15 and 20 °C showed most similar firmness compared to margarine at 20 °C after plasticizing.

The reason for the huge decrease in firmness of the bigels during plasticizing, especially compared to margarine, can be found in the gel-like structure of the oleogel part in the bigels. Subjecting such structures to large deformation, as during the plasticizing step, results in breaks of the gel network strands, which do not recover after plasticizing (Vershkov and Davidovich-Pinhas, 2023; Werner-Cárcamo et al., 2024). In the case of margarine, the firmness is based on the relatively high amount of solid fat and the microstructure of the fat crystal network (Stauffer and Shahidi, 2005; Narine and Marangoni, 1999). Here, structures are also impacted during plasticizing, but the elongated crystallite layers of laminating margarine better dissipate the applied stress, as described by Macias-Rodriguez et al. (2017).

Based on the firmness results after plasticizing, BW18, CBW14, CLW14 and RBW20 were selected for the subsequent baking trials (see 3.2).

In addition to firmness evaluation, the plasticity was evaluated for the selected bigels in comparison to butter and margarine. Table 3 shows the plasticity results at 15 and 20 °C, before and after plasticizing. The plasticity is associated with spreadability and indicates the ability of a fat to keep its shape while still being malleable (Stauffer and Shahidi, 2005). Again, a significant difference is evident between plasticity of all fats before and after plasticizing. The plasticizing step makes the fats more pliable and allows for easier spreading of the fats during lamination (Telloke, 1991b). Plasticity values of selected bigels after plasticizing are in the same range as butter and margarine considering both usage temperatures. These results confirm the selection of suitable wax concentrations as discussed above.

**Table 3:** Plasticity (in Nmm) at different temperatures before or after plasticizing of butter (B), margarine (M) and selected bigels with 18 % beeswax (BW18), 14 % carnauba wax (CBW14), 14 % candelilla wax (CLW14) and 20 % rice bran wax (RBW20) in the oleogel.

	Before plasticizing		After plasticizing	
Fat	15 °C	20 °C	15 °C	20 °C
**B**	62.8 ± 7.3^cd,A^∗		38.8 ± 9.5^abc^,^B^	
**M**		62.7 ± 5.2^cd,A^		45.2 ± 3.6^a,B^
**BW18**	68.4 ± 8.8^bc,A^	49.9 ± 7.7^de,A^	37.3 ± 3.2^bc,B^	27.4 ± 4.2^d,B^
**CBW14**	77.2 ± 9.2^b,A^	70.3 ± 10^bc,A^	36.6 ± 6.6^bc,B^	34.9 ± 3.9^c,B^
**CLW14**	60.5 ± 9.6^cd,A^	48.7 ± 6.4^e,A^	42.9 ± 6.5^ab,B^	34.6 ± 3.8^c,B^
**RBW20**	90.6 ± 10.3^a,A^	77.5 ± 6.3^ab,A^	44.4 ± 5.3^a,B^	31.2 ± 1.9^cd,B^

∗ Different lower-case letters indicate significant differences (p < 0.05) within the same state (before or after plasticizing), different upper-case letters indicate significant differences within the same fat and temperature, but different states.

#### 3.1.2 Solid fat content

The solid fat content (SFC) of fats at different temperatures is often used to predict characteristics like mechanical properties or heat resistance, which correlate with performance in bakery products (Cauvain, 2015; O'Brien, 2004). In this study, SFC values of the control fats, margarine and butter, as well as bigels at different wax concentrations were measured between 10 and 35 °C. Fig. 4A confirms that both margarine and butter have a very steep SFC curve correlating to the higher temperature sensitivity of the mechanical properties of fats as discussed in the previous chapter. On the other hand, only negligible changes in SFC were observed with increasing temperature for the bigel samples (Fig. 4B–E). Measured SFC values were roughly in line with the respective wax concentration in the oleogel or were slightly lower. The highest measured temperature of 35 °C was well below the melting temperatures of the waxes used in this study, which explains the almost constant SFC. Similar results were obtained by Hwang et al. (2022) and Winkler‐Moser et al. (2023). Comparing SFC of bigels with the control fats at temperatures of 10 and 20 °C, where mechanical properties were determined, it is evident that bigels possess much lower SFC than butter and margarine while still having comparable firmness. This is due to the fact that SFC is not solely responsible for the mechanical behaviour, but also the underlaying structuring mechanism (Blake and Marangoni, 2015; Narine and Marangoni, 1999). Conventional fats mainly gain their solid consistency from the amount of material in the solid phase (O'Brien, 2004; Franke et al., 2015). Meanwhile only a little amount of solids, e.g. the wax, is necessary to form a network around the liquid oil to gain a solid consistency in oleogelation (Co and Marangoni, 2012; Doan et al., 2015). Hence, SFC is less useful in predicting the mechanical properties of oleogels or bigels compared to conventional baking fats. In this study, bigels at the same wax concentrations have similar SFC, but can vary distinctly in their mechanical properties. This variation in mechanical properties can be attributed to differences in wax crystal morphology, as further discussed in chapter 3.1.3.

Moreover, SFC data confirmed the lower dependency of bigel firmness on temperature in the range between 10 and 20 °C compared to margarine and butter, as discussed above. From all bigels, the SFC of the ones prepared with BW showed a slightly more pronounced decrease with higher temperature, but it is still much lower than that of margarine or butter. Such a slightly higher temperature dependency might be attributed to the more inhomogeneous composition and lower melting temperature of BW compared to the other waxes (Doan et al., 2017).

#### 3.1.3 Microstructure

**Fig. 5:**
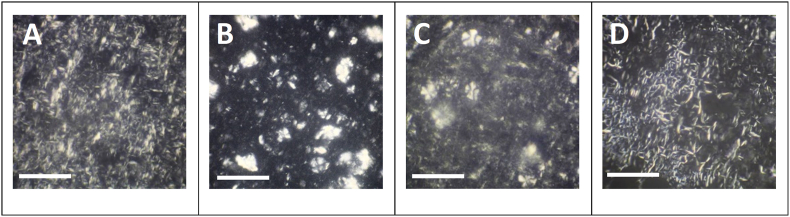
Microstructure of selected bigels. (A) 18 % beeswax, (B) 14 % carnauba wax, (C) 14 % candelilla wax, (D) 20 % rice bran wax in the oleogel. Bar length: 100 μm.

Due to the varying chemical compositions of the selected waxes, they present different crystal morphologies in the bigels after solidification. As can be seen in Fig. 5, crystallized BW and RBW in bigels had a more needle-like morphology, whereas CBW and CLW formed spherulitic crystals. Considering the wax concentration required to obtain the desired texture of the bigels after plasticizing, it seems that the more inhomogeneous spherulitic crystals observable for CBW and CLW resulted in firmer structures. Therefore, lower wax concentrations are necessary. On the other hand, waxes crystallizing in needle-like structures, as BW and RBW, formed weaker structures and higher concentrations are required for the desired texture. Our findings are consistent with data published by Blake et al. (2018) as well as Yılmaz and Öğütcü (2014) about wax crystal morphologies in oleogels. According to Sagiri et al. (2017) the differing morphologies contribute significantly to the respective gelation potential and critical gelling concentrations of the waxes. According to their study, a needle-like or fibrous morphology leads to more efficient gelling compared to spherulitic or platelet-like crystals. This is evident for example in the relatively low critical gelling concentration of RBW of only 1 % compared to CBW, where up to 4 % are necessary for gelling (Blake et al., 2014). However, RBW required the highest concentration of 20 % to achieve the desired high gel firmness in this study, while “only” 14 % of CBW were required (see Fig. 3). This demonstrates that a high gelling efficiency and low critical gelling concentration does not result in a high gel firmness at higher wax concentrations.

### 3.2 Croissants

#### 3.2.1 Dough layer distribution and croissant cross-sections

**Fig. 6:**
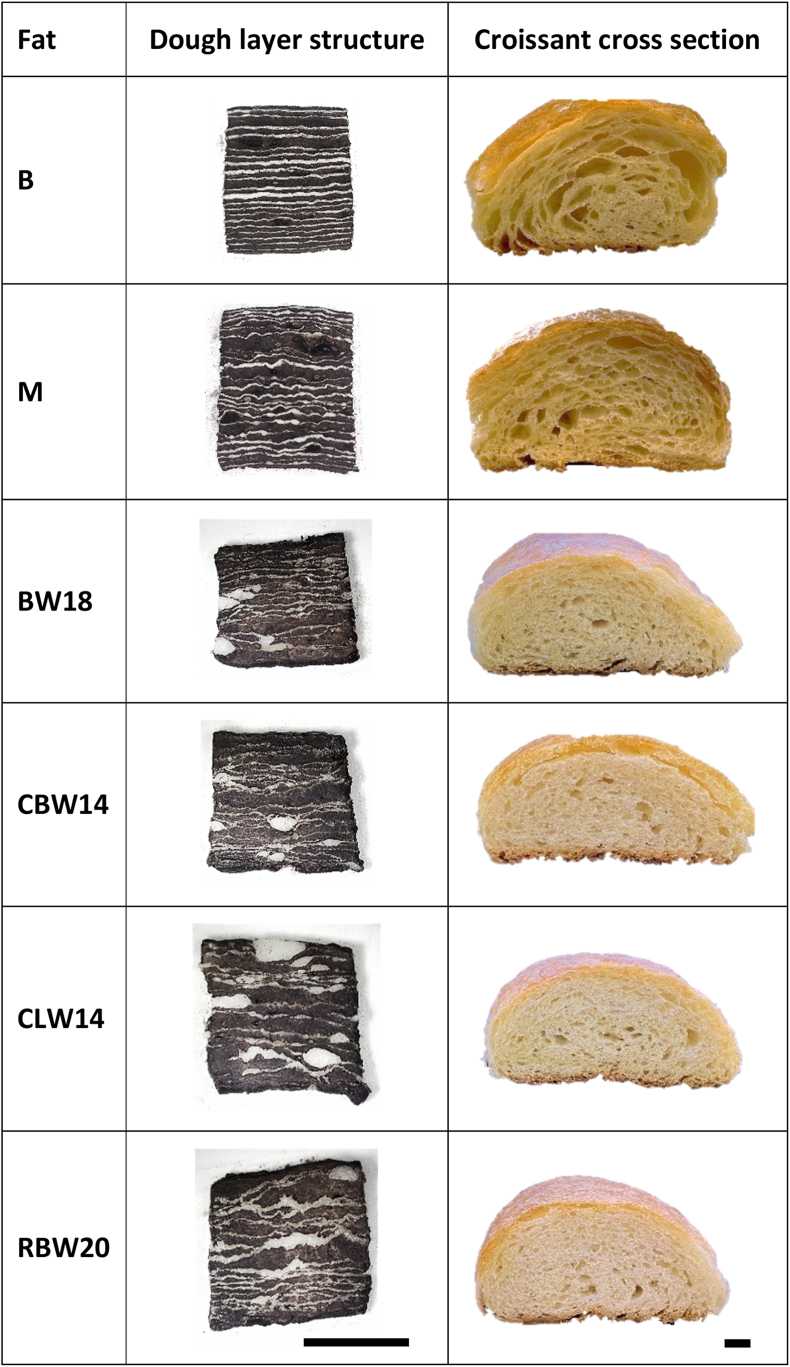
Structure of pastry dough layers (dark: dough, light: fat) and corresponding cross-sections of croissants obtained with control fats margarine (M) and butter (B) as well as selected bigels made with 18 % beeswax (BW18), 14 % carnauba wax (CBW14), 14 % candelilla wax (CLW14) and 20 % rice bran wax (RBW20) in the oleogel. Bar length: 10 mm.

Previously selected bigels BW18, CBW14, CLW14 and RBW20 and the control fats were incorporated into croissant dough and baked. Pictures of the laminated doughs were taken to evaluate the ability of the selected bigels to form the required thin continuous layers. Fig. 6 shows dough cross-sections next to the corresponding cross sections of baked croissants. As mentioned, dough layers were stained using Lugol solution and they appear in dark color whereas fat layers remained white or light grey. Color differences of croissant cross sections are due to differences in lighting and are not related to the fat used.

Butter and margarine doughs display continuous and alternating layers of fat and dough with only slight variations in thickness. Resulting croissant cross sections have a honeycomb-like structure with big air pockets as it is desired for croissants. Although bigel doughs display some continuous fat layers as well, several layers are disrupted and dough layers appear to be fused together. As previously discussed, the repeated mechanical stress put on the bigels during sheeting damages the structure and further softens the gels. If the fat is softer than the dough, it is easily absorbed by the dough and the layering effect is less effective (Stauffer and Shahidi, 2005). Wang et al. (2023) also reported significantly softer laminated doughs when replacing shortening with oleogel-shortening blends. Moreover, doughs with bigels display some thicker fat pieces, which were not fully flattened during sheeting. Similar structures were obtained in a previous study for puff pastry dough (Steinkellner et al., 2024). The irregular lamination resulted in a finer pore structure in the baked bigel croissants. Pore structure will be further described by the specific pore surface area discussed in the next chapter. The disrupted fat layers could not retain the steam during baking to the desired extend. This resulted in a decreased lift and less airy structure with a lower number of larger pores compared to control croissants (Mert and Demirkesen, 2016a; Ghotra et al., 2002). Similarly, a fine croissant pore structure was obtained by Espert et al. (2023) and Wang et al. (2024) when replacing shortenings with hydroxypropyl methylcellulose oleogel-shortening blends. Denser croissants were also obtained when replacing conventional laminating fat with a margarine alternative containing wax oleogel (Blake and Marangoni, 2015).

#### 3.2.2 Croissant quality

Physical properties of croissants made with different laminating fats are summarized in Table 4. The specific volume is an indication for the rise of the croissants in the oven and describes how airy or compact they are. The croissants prepared with the bigels were generally more compact with a lower specific volume by trend. However, in most cases, they did not significantly differ from the butter and margarine croissants. Only croissants prepared with CLW14 had a significantly lower specific volume than the rest of the croissants. Croissants with BW18 had the highest specific volume of all bigel croissants, indicating that they rose the most and had the airiest structure.

**Table 4:** Croissant quality parameters for baking trials using butter (B), margarine (M) or selected bigels made with 18 % beeswax (BW18), 14 % carnauba wax (CBW14), 14 % candelilla wax (CLW14) or 18 % rice bran wax (RBW20) in the oleogel.

Fat	Specific volume (cm^3^/g)	Specific pore surface area (1/cm)	Height expansion (%)	Width expansion (%)
**B**	3.13 ± 0.66^ab^∗	6.62 ± 0.16^c^	16.6 ± 1.71^a^	49.1 ± 4.02^bc^
**M**	3.21 ± 0.36^ab^	7.17 ± 0.63^b^	16.2 ± 1.92^a^	45.4 ± 1.82^c^
**BW18**	3.35 ± 0.09^a^	8.67 ± 0.31^a^	9.38 ± 2.79^b^	56.7 ± 7.27^a^
**CBW14**	2.79 ± 0.34^b^	8.35 ± 0.49^a^	9.77 ± 1.99^b^	55.8 ± 7.71^ab^
**CLW14**	2.16 ± 0.19^c^	8.44 ± 0.48^a^	9.94 ± 2.66^b^	52.9 ± 5.08^abc^
**RBW20**	2.77 ± 0.54^b^	8.58 ± 0.31^a^	10 ± 1.95^b^	46.4 ± 5.93^c^

∗Different letters within columns indicate significant differences (p < 0.05).

This trend is somehow reflected in the specific pore surface area, which is an indicator for the amount and size of pores within the croissants. Again, croissants with BW18 showed the highest specific pore surface area of all bigel formulations, although the differences were not significant. All bigel croissants had a significantly higher specific pore surface area compared to butter and margarine croissants. This means that they had a higher number of small pores, while control croissants had less, but bigger pores. This is also visible in croissant cross sections displayed in Fig. 6.

Height and width expansion were measured to evaluate the degree of vertical and horizontal extension of the croissant pieces after fermentation and baking. This data indicates, whether croissants were able to hold their round shape and rose in height during proofing and baking or if they collapsed and spread out horizontally. Bigel croissants showed significantly lower height expansion compared to butter and margarine croissants, while they generally had higher expansion in width. Due to the high standard deviation for the width expansion, differences were not always significant, but a clear trend can be observed that bigel croissants were generally flatter and wider compared to the control croissants. This trend can be explained by the softer consistency of the bigels and the resulting inconsistent layering discussed in the previous chapter. The very thin dough layers of less than 200 μm did not have much structural integrity and are usually supported by the fat layers (Telloke, 1991a). The bigels used in this study could not support the dough to the same extend. Hence, additional strain was put on the dough during sheeting, which overworked the gluten network. Therefore, the pastries rose at first during fermentation and baking, but the system could not retain its structure and collapsed partially.

#### 3.2.3 Texture profile analysis (TPA)

TPA was performed to gain instrumental data on texture attributes that can be correlated to sensory perception during consumption (Szczesniak, 1963). Szczesniak (1963) defined the firmness (or hardness) as “the force necessary to attain a given deformation”, cohesiveness as “the strength of the internal bonds making up the body of the product”, springiness (or elasticity) as “the rate at which a deformed material goes back to its undeformed condition after the deforming force is removed and chewiness as “the energy required to masticate a solid food product to a state ready for swallowing”. Table 5 displays TPA results of bigel and control croissants on day 0 and day 1, 1 h and 24 h after baking, respectively. In general, bigel croissants did not differ significantly from each other in any texture attribute. Comparing bigel croissants with those prepared from control fats, firmness on day 0 was significantly higher for bigel croissants. In fact, bigel croissants were mostly more than twice as firm as butter and margarine croissants. This can be explained by the much denser pore structure discussed in the previous chapter. Storage for 24 h distinctly increased the firmness of all croissants, independent of the laminating fat. This can be attributed to staling (Gray and Bemiller, 2003). The term staling includes a variety of different processes, some of which are not completely understood yet, but is commonly described as a combination of moisture transfer from crumb to crust and starch retrogradation during storage, which both lead to increased firmness (Cauvain, 2015). As can be observed, the increase in firmness after storage is much higher for the croissants prepared with butter and margarine compared to those with bigels. While butter and margarine croissants had a 3.7- and 3.4-times higher firmness after 24 h of storage respectively, bigel croissant firmness only increased 2.1 to 2.7 times. This could partially be explained by the already higher firmness of bigel croissants after baking. On the other hand, the bigels were formulated with a monoacylglyceride emulsifier, which is known to act as an anti-staling agent in doughs and might contribute to lower increase in firmness (Cauvain, 2015).

**Table 5:** Texture profile data of croissants prepared using butter (B), margarine (M) and selected bigels made with 18 % beeswax (BW18), 14 % carnauba wax (CBW14), 14 % candelilla wax (CLW14) or 20 % rice bran wax (RBW20) in the oleogel.

	Firmness (N)		Springiness (%)		Cohesiveness (%)		Chewiness (−)	
Fat	Day 0	Day 1	Day 0	Day 1	Day 0	Day 1	Day 0	Day 1
**B**	5.16 ± 0.88^c,B^	19.2 ± 1.23^d,A^	85.3 ± 3.44^a,A^	88.8 ± 1.72^a,A^	47.8 ± 4.45^bc,B^	56.8 ± 3.87^a,A^	2.09 ± 0.37^bc,B^	8.16 ± 2.45^bc,A^
**M**	5.84 ± 0.74^c,B^	19.9 ± 1.13^d,A^	52.0 ± 13.2^b,B^	77.8 ± 13.5^a,A^	34.3 ± 2.94^d,B^	48.2 ± 10.7^ab,A^	1.06 ± 0.36^c,B^	7.67 ± 2.81^c,A^
**BW18**	12.2 ± 0.32^ab,B^	26.2 ± 2.00^c,A^	88.5 ± 1.73^a,A^	82.0 ± 4.69^a,B^	60.0 ± 2.94^a,A^	53.3 ± 1.50^ab,B^	6.47 ± 0.28^a,B^	11.5 ± 1.48^b,A^
**CBW14**	13.5 ± 1.52^a,B^	31.1 ± 2.93^ab,A^	86.2 ± 5.34^a,A^	82.0 ± 6.36^a,A^	54.8 ± 4.79^ab,A^	45.8 ± 6.79^b,B^	6.41 ± 1.33^ab,B^	12.0 ± 3.56^a,A^
**CLW14**	10.9 ± 2.17^b,B^	29.5 ± 1.17^bc,A^	75.2 ± 4.31^a,B^	85.5 ± 2.35^a,A^	41.5 ± 7.23^cd,B^	50.7 ± 2.8^ab,A^	3.47 ± 1.36^abc,B^	12.8 ± 0.80^a,A^
**RBW20**	14.2 ± 0.92^a,B^	34.9 ± 4.82^a,A^	86.5 ± 3.87^a,A^	84.0 ± 3.16^a,A^	56.0 ± 3.27^ab,A^	52.5 ± 2.38^ab,A^	6.97 ± 0.91^a,B^	15.4 ± 1.92^a,A^

∗Different lowercase or different uppercase letters indicate significant differences within columns or between day 0 and day 1, respectively (p < 0.05).

Bigel and butter croissants showed comparable springiness on both days. Only margarine croissants exhibited significantly lower springiness on day 0. Similar behaviour was observed for cohesiveness. This indicates that the structure of the croissants with margarine was more severely damaged. In fact, the margarine croissants were crunchier than butter and bigel croissants. After the first compression, the crunchy outer layer of the margarine croissants was broken leading to the lower springiness and cohesiveness values.

Croissant chewiness is calculated from the three previously discussed texture attributes. Unsurprisingly, bigel croissants exhibited higher chewiness compared to control croissants and after over-night storage chewiness significantly increased for all fats. Those croissants therefore have a higher internal resistance to mechanical deformation or destruction.

In the studies by Espert et al. (2023) and Wang et al. (2024), TPA was performed on croissants made with hydroxypropyl methylcellulose-based oleogels blended with shortening at different ratios. They performed the test 24 h after baking similarly to day 1 in the present study. Increased croissant cohesiveness and chewiness were measured with increasing oleogel concentrations, whereas springiness of the oleogel croissants did not differ from that of the control samples. These findings are consistent with the results of the present study. However, they did not find significant differences in firmness between shortening and oleogel croissants, even though they also observed denser pore structures, which have been shown to increase pastry firmness (Jung et al., 2020). Oppositely, increased firmness was determined in the present study for bigel croissants. These differences between the studies could be attributed to the hydrogel part of the bigels, whereas no water is present in the oleogel. Since the bigel structure is impacted during lamination, both water and oil might be absorbed by the dough affecting its gluten network and structure. While oil interferes with the development of the gluten network, water facilitates this process (Cauvain and Young, 2008). The increased cross-linking of the protein network in the bigel-doughs could therefore explain the increased croissant firmness in comparison to the water-free oleogel.

#### 3.2.4 Saturated fatty acid content

Based on nutritional information provided by the suppliers, the butter and margarine used contained 82 % and 80 % fat, transferring to a SFA content of 56 % and 42 %, respectively. Similarly, the bigels consist of 80 % oleogel phase, which contains, depending on the used wax concentration, approximately 80 % canola oil, which only has 6.5 % SFA. Even though waxes would have considerable contents of SFA, they are poorly absorbed in the digestive tract of mammals and can be excluded from the calculation of nutritional data (Doan et al., 2017; Hargrove et al., 2004). Including the minor contribution of the monoacylglyceride emulsifier, the final bigels have a nutritionally relevant SFA content of 5.1–5.4 %. This results in a reduction of almost 90 % SFA in the bigel, and, therefore, also in the baked croissants compared to the conventional butter and margarine used in this study.

## 4 Conclusion

This study investigated the characteristics of bigels made from xanthan-based hydrogels and canola oil oleogels with beeswax, carnauba wax, candelilla wax and rice bran wax as oleogelators at varying concentrations. Bigel properties were investigated with respect to their suitability as laminating fats in comparison to commercial butter and margarine in order to reduce the saturated fatty acid content in croissants. Wax concentrations much higher than usually investigated were necessary to gain the required consistency. The significant reduction in firmness after large deformation during plasticizing highlights the importance of extensive optimization experiments and measuring the influence of deformation on bigel properties before usage in food products. Incorporating the bigels into croissant dough still presents some challenges, resulting in pastries with slightly lower quality compared to pastries made from margarine or butter, whereas the nutritional profile can be considerably improved by the use of bigels.

In the future, combinations of different oleogelators and/or a modified hydrogel composition should be investigated to lower the required gelator amount. Furthermore, upscaling of the bigel production process, considering cooling rates and possibly cooling under shear shall be studied to improve the bigel consistency and its stability during the lamination process for application in laminated doughs. Sensory testing will also be necessary to inquire about consumer acceptance of the bigels as a fat alternative in laminated pastries.

## CRediT authorship contribution statement

**Christine Steinkellner:** Conceptualization, Methodology, Data curation, Investigation, Formal analysis, Visualization, Writing – original draft, Writing – review & editing. **Lina Kroll:** Investigation, Formal analysis. **Knut Franke:** Supervision, Funding acquisition, Writing – review & editing.

## Declaration of competing interest

The authors declare that they have no known competing financial interests or personal relationships that could have appeared to influence the work reported in this paper.

## Data Availability

Data will be made available on request.
